# New Polymeric Hydrogels with Cannabidiol and α-Terpineol as Potential Materials for Skin Regeneration—Synthesis and Physicochemical and Biological Characterization

**DOI:** 10.3390/ijms25115934

**Published:** 2024-05-29

**Authors:** Martyna Zagórska-Dziok, Anna Nowak, Anna Zgadzaj, Ewa Oledzka, Karolina Kędra, Agnieszka Ewa Wiącek, Marcin Sobczak

**Affiliations:** 1 Department of Technology of Cosmetic and Pharmaceutical Products, Faculty of Medicine, University of Information Technology and Management in Rzeszow, 2 Sucharskiego St., 35-225 Rzeszow, Poland; mzagorska@wsiz.rzeszow.pl; 2 Department of Cosmetic and Pharmaceutical Chemistry, Pomeranian Medical University in Szczecin, 70-111 Szczecin, Poland; anowak@pum.edu.pl; 3 Department of Environmental Health Sciences, Faculty of Pharmacy, Medical University of Warsaw, 1 Banacha St., 02-097 Warsaw, Poland; anna.zgadzaj@wum.edu.pl; 4 Department of Pharmaceutical Chemistry and Biomaterials, Faculty of Pharmacy, Medical University of Warsaw, 1 Banacha Str., 02-097 Warsaw, Poland; eoledzka@wum.edu.pl; 5 Institute of Physical Chemistry, Polish Academy of Sciences, 44/52 Kasprzaka St., 01-224 Warsaw, Poland; kkedra@ichf.edu.pl; 6 Department of Interfacial Phenomena, Faculty of Chemistry, Maria Curie-Skłodowska University, pl. Sq. M. Curie-Skłodowskiej 3, 20-031 Lublin, Poland; agnieszka.wiacek@mail.umcs.pl

**Keywords:** transdermal active substance delivery systems, hydrogels, biodegradable polymers, cannabidiol, α-terpineol, antioxidants, metalloproteinase inhibitors, antimicrobial activity, cytotoxicity, skin cells

## Abstract

Dermatology and cosmetology currently prioritize healthy, youthful-looking skin. As a result, research is being conducted worldwide to uncover natural substances and carriers that allow for controlled release, which could aid in the battle against a variety of skin illnesses and slow the aging process. This study examined the biological and physicochemical features of novel hydrogels containing cannabidiol (CBD) and α-terpineol (TER). The hydrogels were obtained from ε-caprolactone (CL) and poly(ethylene glycol) (PEG) copolymers, diethylene glycol (DEG), poly(tetrahydrofuran) (PTHF), 1,6-diisocyanatohexane (HDI), and chitosan (CHT) components, whereas the biodegradable oligomers were synthesized using the enzyme ring-opening polymerization (e-ROP) method. The in vitro release rate of the active compounds from the hydrogels was characterized by mainly first-order kinetics, without a “burst release”. The antimicrobial, anti-inflammatory, cytotoxic, antioxidant, and anti-aging qualities of the designed drug delivery systems (DDSs) were evaluated. The findings indicate that the hydrogel carriers that were developed have the ability to scavenge free radicals and impact the activity of antioxidant enzymes while avoiding any negative effects on keratinocytes and fibroblasts. Furthermore, they have anti-inflammatory qualities by impeding protein denaturation as well as the activity of proteinase and lipoxygenase. Additionally, their ability to reduce the multiplication of pathogenic bacteria and inhibit the activity of collagenase and elastase has been demonstrated. Thus, the developed hydrogel carriers may be effective systems for the controlled delivery of CBD, which may become a valuable tool for cosmetologists and dermatologists.

## 1. Introduction

Skin diseases are a significant problem affecting people worldwide [1]. In these diseases, there are various skin lesions within individual layers of the skin, often accompanied by persistent itching, burning of the skin, and soreness [2]. Due to many limitations of currently available therapies, new solutions are still being sought, including new carriers of active substances that could help rise to this challenge. Treatments involving the topical application of medicinal substances are being developed globally due to the multiple systemic adverse effects of oral therapies that are routinely used to treat skin problems [3]. Numerous attempts have been made to develop various drug carriers that would enable increased penetration of biologically active substances into the skin, contributing to the surface effect of medicinal substances and improving transdermal therapy [4].

Hydrogels are promising carriers of natural compounds that can significantly improve the treatment effectiveness of skin diseases. These drug delivery systems (DDSs) are three-dimensional polymer networks into which many therapeutic compounds, both hydrophilic and hydrophobic, can be incorporated, which allows them to be used in the treatment of a wide range of diseases [5,6]. The ability to design smart hydrogels that can release embedded substances in a controlled manner in response to changing stimuli allows for control of the release rate of therapeutic compounds from these three-dimensional networks [7]. Therefore, hydrogel carriers are one of the most dynamically developing groups of medical materials, and their use in cosmetology and dermatology is constantly increasing. Their remarkable advantage is the ease of application and significant minimization of side effects associated with the topical method of drug application, leading to therapeutic concentrations in individual layers of the skin without increasing the concentration in the serum [8]. Hydrogels based on natural polymers, characterized by high biocompatibility, biodegradability, and low toxicity and immunogenicity, are currently gaining great popularity [9]. Their physical properties are similar to natural tissues, and the constant development of various chemical and physical strategies for their synthesis led to obtaining biofunctional hydrogels with the desired properties in treating specific skin diseases [10].

The use of hydrogels in the treatment of various dermatological diseases and in improving the condition of the skin focuses primarily on their use as carriers with a highly controlled release of active substances in treating local skin lesions, in tissue engineering, regenerative medicine, and cell therapy [6,11,12]. The enormous potential of active, natural-origin substances used to treat many skin diseases has been described in scientific reports for a long time. Natural compounds assessed in the context of their therapeutic application in the treating of skin diseases are analyzed primarily in terms of their antioxidant, anti-inflammatory, and antimicrobial activity [13]. In order to assess the safety of their use, cytotoxicity and genotoxicity tests are also carried out [14].

A promising compound in treating various skin diseases is cannabidiol (CBD), which has a comprehensive therapeutic effect [15]. Because the endocannabinoid system is a fundamental molecular system responsible for controlling and maintaining homeostasis in the body, it is nowadays a common target of pharmacotherapy [16]. After discovering the endocannabinoid system of the skin and its essential role in maintaining the homeostasis of this organ, interest in phytocannabinoids increased significantly [17]. CBD, by interacting with cannabinoid receptors and other elements of the endocannabinoid system, may contribute to achieving satisfactory treatment effects related to its multidirectional action [16]. This compound may be beneficial in the treatment of skin diseases because it has strong antioxidant properties and can also inhibit the inflammatory processes that often accompany this type of disorder [16]. It also has an analgesic, antibacterial, and neuroprotective effect, which may contribute to better achievement of therapeutic results [18,19,20,21]. Moreover, it has also been confirmed that CBD has a protective effect, including the integrity of skin cell membranes [16]. Preclinical studies indicate that the topical application of this compound may be practical in treating certain skin conditions such as psoriasis, eczema, pruritus, and inflammation [22]. It has also been suggested that it may prove helpful in treating skin cancers, mainly those developing due to a long-term inflammatory process [23,24].

A valuable compound with a broad spectrum of activity is also the natural monoterpene α-terpineol (TER), which occurs naturally in flowers, herbs, and essential oils [25]. Its use in the treatment of various diseases is constantly increasing due to its antioxidant and anticancer properties [26,27]. It is also seen as an anticonvulsant, antibacterial, antihypertensive, and anti-ulcer substance [25,26,28]. Moreover, it has been proven that this compound increases skin penetration. Hence it is often used as a compound that increases the penetration of various biologically active substances, thus increasing the effectiveness of treatment [29,30].

The volatile components of essential oils, such as TER, can penetrate the individual layers of the skin, which contributes to the greater penetration of various drugs from topical preparations into the lower layers of the skin [29]. So far, various mechanisms of action of these compounds have been described, contributing to the increased penetration of the therapeutic substances. One of the mechanisms involves the breakdown of the intercellular lipid structure found between the corneocytes in the stratum corneum [29,31]. Increased penetration may also be closely related to the interaction of these compounds with the intracellular domain of proteins, which may induce a change in their conformation [32,33]. Additionally, this increase may also be due to an increase in drug partitioning [34]. A crucial aspect affecting the safety of their use is that when applied to the skin, the components of essential oils are quickly metabolized, preventing their accumulation in the body and resulting in their rapid excretion [29]. Thus, using TER and other essential oil components may be preferred over various commonly used synthetic materials and be viewed as safe penetration and percutaneous absorption enhancers for both hydrophilic and lipophilic drugs [30,35]. Moreover, CBD, due to its lipophilic nature, can accumulate in cell membranes and thus exhibit antioxidant activity on membrane components for extended periods [16]. Thus, incorporating two active compounds, CBD and TER, into hydrogel materials seems to be a way to improve the effectiveness of topical preparations used in dermatology and cosmetology. Therefore, to ensure the multidirectional effect of developed hydrogel matrices, a combination of CBD and TER was selected to give hydrogels both a broad biological activity related to the presence of CBD and to increase the ability of this compound to penetrate the individual layers of the skin. The purpose of using a penetration enhancer of natural origin was to additionally contribute to increasing the biological activity of this DDS. Besides increasing penetration, TER is a biologically active compound that can support the therapeutic effect of developed CBD matrices. Considering the properties mentioned above of active compounds incorporated into the structure of the developed hydrogels, our DDSs could help in the treatment of the target diseases atopic dermatitis, acne, psoriasis, and skin allergies. Due to the ability of CBD to act on the endocannabinoid system, these hydrogels may be helpful in the treatment of autoimmune diseases, and the antioxidant, anti-inflammatory and antibacterial properties of these compounds may support the treatment of skin diseases accompanied by inflammation and bacterial infections. In this study, we report the synthesis of new hydrogels derived from ε-caprolactone (CL) and poly(ethylene glycol) (PEG) copolymers, diethylene glycol (DEG), poly(tetrahydrofuran) (PTHF), 1,6-diisocyanatohexane (HDI), and chitosan (CHT) as potential materials for the regeneration and treatment of skin diseases. CBD and TER have been used as natural active substances. The main goal of the research was to develop new dermal DDSs characterized by highly controlled active substances released without a “burst release” phase. We were the first to develop a hydrogel biomaterial from the above-mentioned raw materials. The rate of the active ingredients’ release from the synthesized DDSs was investigated in vitro. The obtained hydrogels are suspected to have controlled active substance release profiles.

The developed hydrogels and incorporated active compounds were also assessed regarding their biological properties, including antioxidant, anti-inflammatory, anti-aging, and antimicrobial properties. Additionally, the cytotoxicity of the developed DDS on keratinocytes and fibroblasts was determined. 

## 2. Results and Discussion

### 2.1. Hydrogel Preparation and Characterization

**Figure 1:**
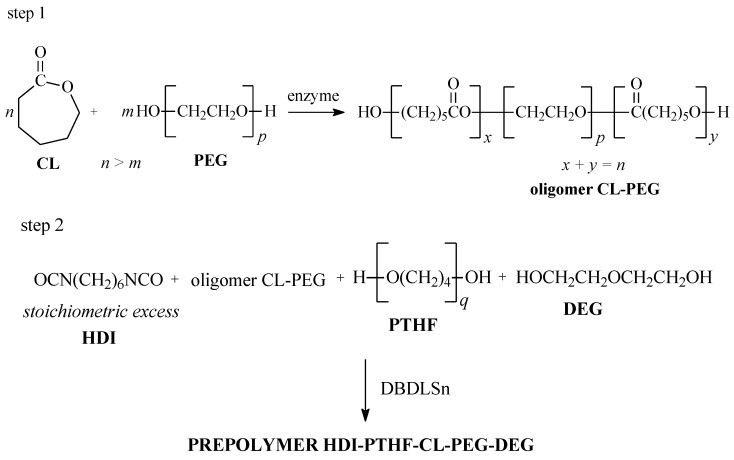

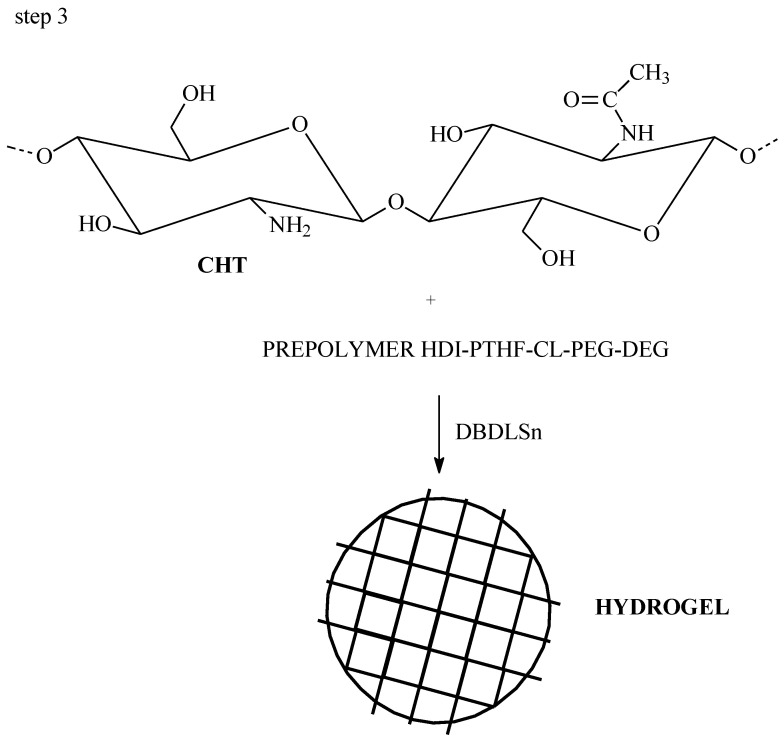
Scheme of the hydrogel CHT-HDI-DEG-PTHF-CL-PEG-DEG synthesis.

Hydrogels were obtained in a three-step method through the reaction of isocyanate-terminated polyurethane (PU) prepolymer and CHT (CHT-HDI-DEG-PTHF-CL-PEG-DEG) (Figure 1). Similar biomaterials were obtained as described in our previous work but using different substrates [11].

In the first step, CL-PEG copolymers were synthesized via the enzyme ring-opening polymerization process (e-ROP) (Table 1). As is commonly known, the ROP of cyclic esters is generally carried out in the presence of metal-containing catalysts. This may pose some toxicological risks and thus reduce the biosafety of using the obtained biomaterial. Therefore, we decided to use the enzyme as a biocatalyst of the ROP process. The obtained polyesters did not require additional purification before being used to synthesize prepolymers and hydrogels. Moreover, the hydrolytic purification process leads to a reduction in the average molar mass of the obtained polyesters. Our goal was to obtain oligomers with a number average molar mass (*M_n_*) in the range of 3000–3500 g/mol. Based on the preliminary research, it was found that this is the optimal molar mass for oligomers used for the synthesis of CHT-PU hydrogels. The molar ratio of CL to PEG was 30:1, 40:1, 50:1, or 60:1. The enzyme ring-opening polymerization e-ROP of CL and PEG was carried out in the presence of immobilized lipase B from *Candida antarctica* (CA). The synthesized copolymers were characterized by *M_n_* = 2400–3600 g/mol. The reaction yields ranged from 71.0 to 92.0%.

After that, the CL-PEG C copolymer was used as a raw material for the hydrogel synthesis because it was characterized by the optimal molar mass and the lowest *Đ* value.

The prepolymers of HDI, CL-PEG copolymer, PTHF, and DEG were obtained in the second step. In the final step, CHT-HDI-DEG-PTHF-CL-PEG-DEG hydrogels were prepared. The prepolymers were obtained in the polyaddition process of HDI, CL-PEG copolymer, PTHF, and DEG in a molar ratio of 2.1:0.7:0.2:0.1. Finally, the hydrogel was reacted with CHT in an NCO (prepolymer)/OH (or NH_2_) (CHT) ratio of 1.65:1 at 80.0 °C for 4 h under an argon atmosphere. Dibutyltin dilaurate (DBDLSn) was used as a polyaddition catalyst.

The obtained hydrogels had the form of white or gray, flexible solids. Their structure was stable at room temperature. The swelling capacity of the obtained CHT-HDI-DEG-PTHF-CL-PEG-DEG hydrogel was determined. The value of the coefficient of the mass swelling ratio (MSR) was 384.0, 445.0, 492.0, and 509.0% after 2 h, 4 h, 8 h, and 24 h, respectively.

### 2.2. In Vitro Release Studies of CBD and TER from Hydrogels

**Figure 2:**
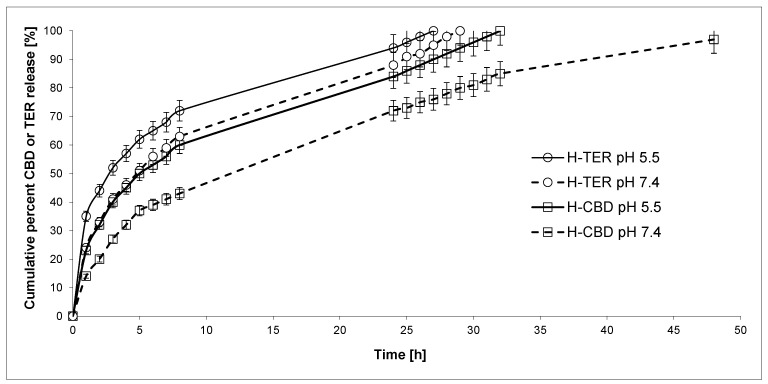
CBD and TER release profiles from the obtained H-CBD and H-TER hydrogels (at pH 5.5 and 7.4) (each point represents the mean ± SD of three points).

**Figure 3:**
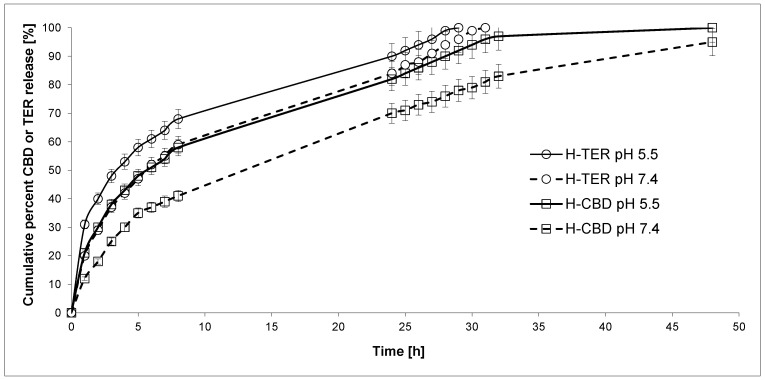
CBD and TER release profiles from the obtained H-CBD-TER hydrogel (in pH 5.5 and 7.4) (each point represents the mean ± SD of three points).

Next, DDSs were obtained from previously synthesized CHT-HDI-DEG-PTHF-CL-PEG-DEG hydrogels. Our goal was to investigate the kinetics of the release of active substances from them. To our knowledge, obtained DDSs are the first of this type of CBD and TER carriers. We were interested in the rate at which active substances with different hydrophilic–hydrophobic properties would be released from hydrogels. CBD and/or TER was/were loaded into hydrogels using the incorporation method. The mean weight of the devices developed was approximately 0.45 g, containing 5.0% CBD (H-CBD), TER (H-TER), or 2.5% CBD and 2.5% TER (H-CBD-TER). In vitro studies of the release of active substances from the obtained hydrogel materials were determined at pH 7.4, 5.5, and 37.0 °C for 48 h (Figure 2 and Figure 3). The plot’s ordinate was calculated based on the cumulative amount of CBD and/or TER released concerning its/their initial amount in the hydrogels.

Generally, TER was released faster than CBD from the obtained hydrogels, both at pH 7.4 and at pH 5.5. This was expected because TER is more soluble in the acceptor liquid. The findings imply that the rate of CBD and TER release from the hydrogels increases as the pH of the solution decreases. For example, the percentage of the released CBD after 24 h of incubation was about 72.0% from the H-CBD hydrogel at pH 7.4 and 84.0% at pH 5.5. For comparison, 88.0% and 94.0% of TER was released from H-TER at 7.4 and 5.5, respectively. A similar result was obtained for the hydrogel containing both active substances (CBD and TER). After 24 h, 70.0% CBD and 84.0% TER at pH 7.4 and 82.0% CBD and 90.0% TER at pH 5.5 were released from H-CBD-TER hydrogel.

The data points obtained for the CBD and/or TER release studies were subjected to zero- and first-order kinetics and the Korsmeyer–Peppas models to evaluate the kinetics and mechanism of active substance release from the hydrogel materials (Table 2). As is known from the literature, according to the Korsmeyer–Peppas model, for the diffusion-degradation controlled drug release system, the release exponent value *n* is in the range of 0.45 and 0.89 (anomalous, non-Fickian). In contrast, when *n* is close to 0.45, the diffusion (Fickian diffusion) predominates in the process, and, in the opposite case, *n* > 0.89, the model corresponds to the super case II transport [36].

The CBD release kinetic at pH 7.4 and pH 5.5 from H-CBD and H-CBD-TER followed the near-first-order model (*R*^2^ = 0.918–0.972). Furthermore, it was noted that TER was released at pH 7.4 and pH 5.5 from H-TER and at pH 5.5 from H-CBD-TER with rather first-order kinetics (*R*^2^ = 0.897–0.971). In turn, when the release process was conducted at pH 7.4, it was observed that for H-CBD-TER, the TER release was close to the near-zero-order kinetics (*R*^2^ = 0.915). The analysis of CBD release data using the Korsmeyer–Peppas model suggested that H-CBD and H-CBD-TER hydrogels at pH 7.4 were governed instead by non-Fickian transport (*n* was 0.503 and 0.511, respectively). In the case of H-TER, H-CBD-TER at pH 7.4, and all hydrogels at pH 5.5, the active substances were released according to the Fickian transport mechanism. It should be noted, however, that the R^2^ coefficients were sometimes below 0.8. Therefore, the obtained results should be treated with some approximation. In our opinion, the obtained biomaterials are characterized by relatively high control over the release of active substances. Nevertheless, optimization studies should be carried out in the future. These studies will certainly allow the determination of the relationship between the structure of the hydrogel and the kinetics of the release of CBD and TER.

### 2.3. Erosion Studies of Hydrogels

**Figure 4:**
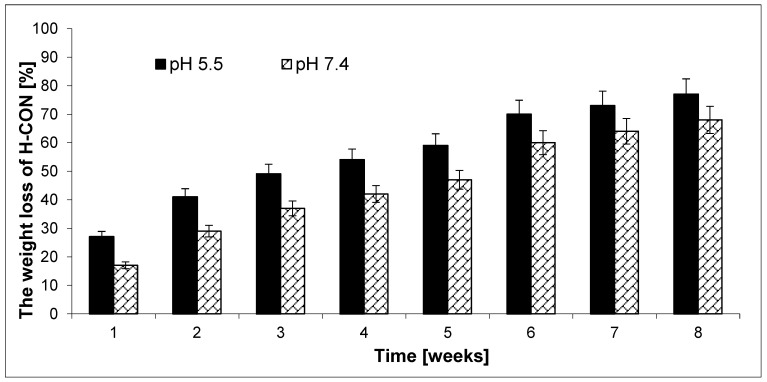
The weight loss of the H-CON during 8 weeks at pH 7.4 and 5.5 (each point represents the mean ± SD of three points).

Since each biomaterial should be safe for humans and the environment after completion of therapy, we decided to investigate the kinetics of decomposition of the obtained hydrogels. The erosion test of the CL-PEG C hydrogel obtained without an active substance (H-CON) was carried out. The test of the resulting hydrogels was conducted under the same conditions as the CBD and/or TER release experiments. The erosion process was characterized by plotting the hydrogel’s weight loss (*WL*) versus time (Figure 4). It was found that the erosion rate of H-CON depends on the pH of the solution. Hydrogels degraded faster at pH 5.5 than at pH 7.4, which was consistent with the CBD and/or TER release experiment data.

The *WL* value for H-CON was 77.0% at pH 5.5 after 8 weeks of erosion testing. In turn, the *WL* value for H-CON was 68.0% at pH 7.4 after 8 weeks of degradation. The results of the tests performed over 8 weeks are satisfactory. Generally, the erosion process of the obtained hydrogels was relatively regular.

### 2.4. Assessment of Antioxidant Properties

The antioxidant properties of the tested hydrogels were assessed using DPPH and ABTS radicals. Furthermore, their impact on free radical levels inside fibroblasts and keratinocytes was investigated. The results indicated the concentration-dependent strong antioxidant properties of H-CBD and H-CBD-TER hydrogels, confirmed by DPPH and ABTS assays. In the first one, the level of free radical scavenging for H-CBD and H-CBD-TER hydrogels was over 80.0%, while in the second, it exceeded 90.0% (Table 3). Different results were observed for the H-TER hydrogel, for which the DPPH test indicated no antioxidant properties. In contrast, the ABTS test showed the possibility of inhibiting this radical at a low level, reaching 20.0% in the case of the highest concentration (5.0%) used (Table 3).

**Figure 5:**
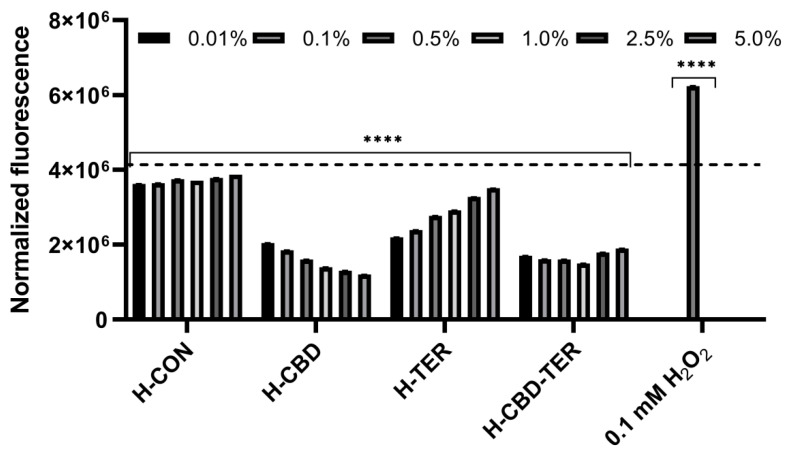
Effect of tested hydrogels on the intracellular level of ROS in fibroblasts (BJ cells). The dashed horizontal line in the figure indicates the fluorescence intensity of the control cells (untreated with hydrogels). **** *p* < 0.0001 versus the control.

**Figure 6:**
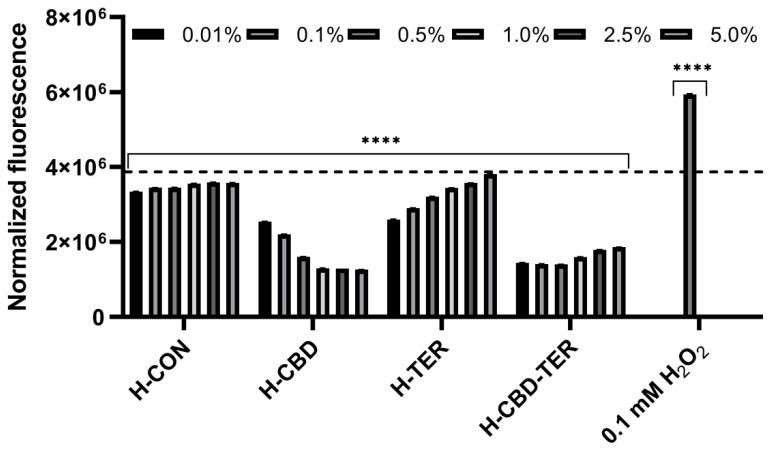
Effect of tested hydrogels on the intracellular level of ROS in keratinocytes (HaCaT cells). The dashed horizontal line in the figure indicates the fluorescence intensity of the control cells (untreated with hydrogels). **** *p* < 0.0001 versus the control.

The test performed to assess the intracellular level of free radicals (ROS) using the fluorogenic H_2_DCFDA dye also showed significant differences in the antioxidant activity of the hydrogels tested. It was observed that the most beneficial effect, both in the case of keratinocytes (HaCaT) and fibroblasts (BJ), is demonstrated by the H-CBD hydrogel, which significantly reduces the level of ROS in both types of cells. As the concentration of this hydrogel increases, the level of radicals decreases. A significant reduction in the amount of free radicals compared to the control was also observed for the H-CBD-TER hydrogel. In contrast, the effect was weaker for this hydrogel as the concentration increased. In the case of the H-TER hydrogel, a decrease in the intracellular level of ROS in BJ and HaCaT cells was also observed. However, as the concentration of this hydrogel increases, the level of ROS increases, but even at the highest concentration tested, it does not reach the level of control cells (Figure 5 and Figure 6).

**Figure 7:**
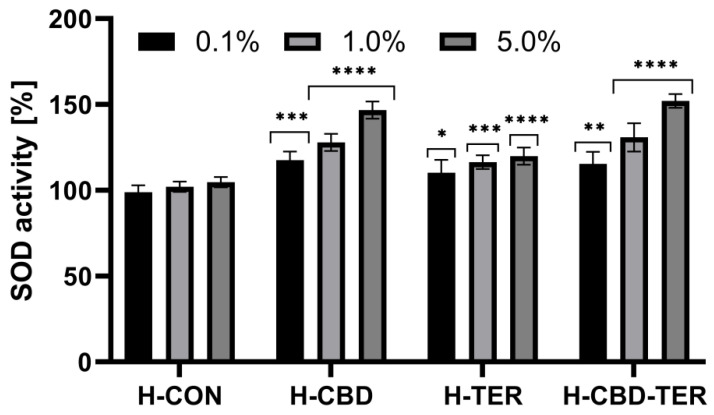
The effect of hydrogels on the activity of superoxide dismutase. **** *p* < 0.0001, *** *p* ≤ 0.0009, ** *p* = 0.0017, * *p* = 0.0476 versus the control.

The study also assessed the influence of the analyzed hydrogels on the activity of the enzyme superoxide dismutase (SOD), which is a crucial element of the antioxidant barrier that protects cells against the toxic effects of free oxygen radicals. The obtained results indicate that hydrogels with CBD, as well as CBD and TER, can significantly increase this enzyme’s activity in vitro. At the same time, materials with TER show a less noticeable effect. When using a 5.0% concentration of hydrogels, an increase in SOD activity was achieved to 146.0% for H-CBD and 152.0% for H-CBD-TER (Figure 7).

The antioxidant properties of CBD and TER observed in this study confirm numerous scientific studies that assessed these properties [16,37,38]. This activity is significant in designing hydrogel carriers and other delivery systems of active substances intended for treating dermatological diseases because the excessive production of free radicals leads to many skin diseases and also significantly affects skin appearance [39]. So far, many of CBD’s antioxidant activity mechanisms have been described. One of them assumes that CBD reduces oxidative stress by preventing superoxide radical formation, mainly caused by xanthine oxidase and NADPH oxidases, both NOX1 and NOX4 [40,41]. As shown by Borges and da Silva, the antioxidant potential of CBD may be related to the abstraction of electrons and hydrogen [38]. This cannabinoid also affects redox balance by being able to alter the level and activity of both antioxidants and oxidants [16]. CBD also reduces ROS production by chelating transition metal ions involved in the Fenton reaction [42]. The antioxidant activity of this compound is also associated with activating a redox-sensitive transcription factor called nuclear factor erythroid 2 (NRF2), which is responsible for the transcription of antioxidant genes with cytoprotective properties [43]. Studies also show that this compound increases the level of superoxide dismutase (SOD) mRNA and increases the enzymatic activity of Cu-, Zn-, and Mn-SOD, responsible for the metabolism of superoxide radicals [41]. Moreover, it can form adducts with glutathione and cytochrome P450 3A11, thus reducing their biological activity [44]. The antioxidant activity of this compound is also associated with supporting the activity of antioxidant enzymes by preventing the decrease in the level of micronutrients necessary for the proper biological activity of enzymes such as superoxide dismutase or glutathione peroxidase [45]. CBD’s antioxidant activity is also associated with scavenging free radicals, breaking their chain reactions, and transforming them into less active forms [16]. A significant effect of the antioxidant activity of CBD is also the reduction in lipid and protein modification [16,45]. We have shown in this study that the antioxidant properties of TER are significantly weaker than those of CBD and other conventionally used antioxidants, which was also confirmed in scientific articles by other authors [46]. However, the incorporation of both of these compounds into the structure of the hydrogel materials shows a strong antioxidant potential related to the possibility of scavenging ROS, increasing the activity of SOD, and reducing the intracellular level of ROS in skin cells in vitro.

### 2.5. Assessment of Cytotoxicity

**Figure 8:**
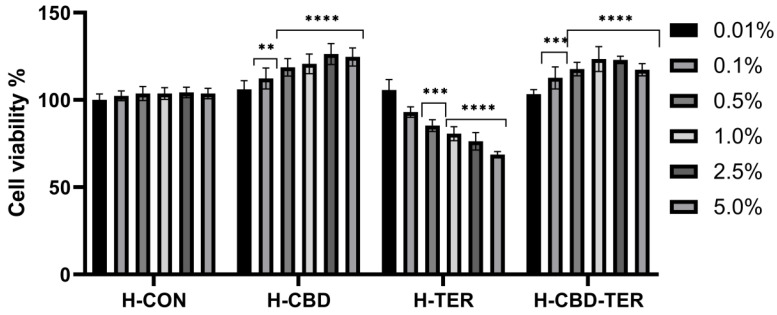
Reduction of resazurin after 24 h of exposure to hydrogel extracts (0.01–5.0%) in cultured fibroblasts. **** *p* < 0.0001, *** *p* ≤ 0.001, ** *p* = 0.0013 versus the control (100%).

**Figure 9:**
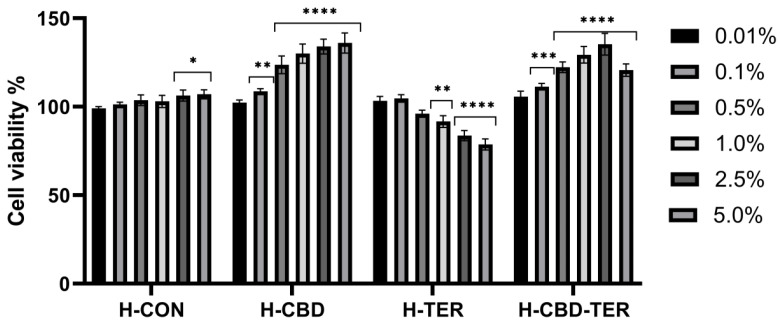
Reduction of resazurin after 24 h of exposure to hydrogel extracts (0.01–5.0%) in cultured keratinocytes. **** *p* < 0.0001,*** *p* = 0.0007, ** *p* = 0.0058, * *p* ≤ 0.0493 versus the control (100%).

**Figure 10:**
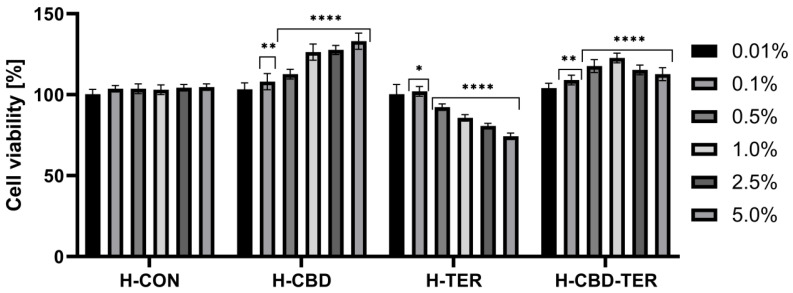
Effect of various concentrations of hydrogel extracts (0.01–5.0%) on NR uptake in cultured fibroblasts after 24 h of exposure. **** *p* ≤ 0.0001, ** *p* ≤ 0.0071, * *p* = 0.0104 versus the control (100%).

**Figure 11:**
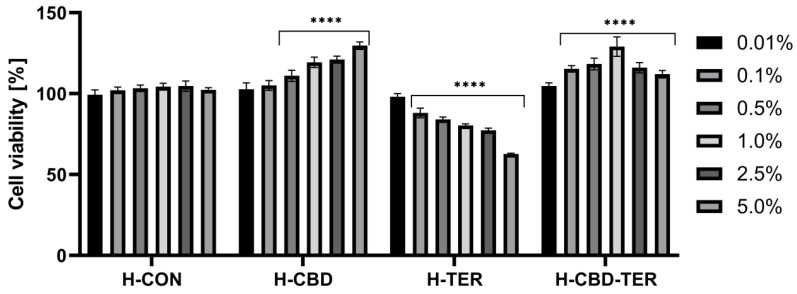
Effect of various concentrations of hydrogel extracts (0.01–5.0%) on NR uptake in cultured keratinocytes after 24 h of exposure. **** *p* < 0.0001 versus the control (100%).

As part of the analyses, the tested hydrogels’ effect on cell membranes’ metabolic activity and integrity was assessed using the resazurin assay (AB assay) and the neutral red assay (NRU assay). Cytotoxicity was assessed on cells constituting various layers of the skin, BJ and HaCaT. The obtained results indicated significant differences in cytotoxicity between the tested hydrogels. This cytotoxicity was concentration-dependent. In the case of the AB test, a positive effect of H-CON and H-CON-TER hydrogels was noted, for which an increase in the viability of fibroblasts was achieved to 126.0% and 123.0%, respectively. In the case of HaCaT cells, the increase in viability reached 136.0% for H-CBD and 135.0% for H-CBD-TER. Only in the two lowest concentrations (0.01 and 0.1%) did H-TER hydrogel not induce cytotoxicity on BJ and HaCaT cells. In contrast, with the concentration increasing, their viability decreased to 69.0% and 78.0%, respectively (Figure 8 and Figure 9). Similar results were obtained in the NRU test. This test showed that with an increase in the concentration of the H-CBD hydrogel, the viability of both cell lines increases to about 130.0% when using a 5.0% hydrogel concentration. For the H-CBD-TER hydrogel, the most positive effect was observed for the concentration of 1.0%, for which an increase in viability was observed by 126.0% for BJ cells and 129.0% for HaCaT. The H-TER hydrogel showed a dose-dependent cytotoxic effect on both cell types, limiting their viability to 74.0% (BJ cells) and 63.0% (HaCaT cells) (Figure 10 and Figure 11).

Assessing the impact of DDSs to be applied on the skin surface is critical in assessing their safety. The research results described above indicate that the hydrogels H-CBD and H-CBD-TER do not induce cytotoxic effects on skin cells in vitro, which may indicate their potential use in the treatment of skin diseases. Casares et al. pointed out the crucial role of CBD in the differentiation of keratinocytes, skin development, and differentiation of epidermal cells. They showed that CBD can induce keratinocyte proliferation, expression of several NRF2 target genes, nuclear export and degradation of BACH1, expression of heme oxygenase 1 (HMOX1) independently of NRF2, and increases in keratin 16 and 17 levels, which significantly affects keratinocyte proliferation and wound repair [47]. Sangiovanni et al. noted that the positive effect of CBD on skin cells is also associated with the inhibition of the release of inflammatory mediators by impairment of the kappa B (NF-κB) nuclear pathway, which was observed in both keratinocytes and fibroblasts. This compound also influences the release of interleukin-8, vascular endothelial growth factor (VEGF), and metalloproteinase 9 (MMP-9) [48]. Studies by Atalay et al. also indicate that CBD effectively protects keratinocytes against the adverse effects of UVB radiation, which is mainly related to the change in the expression of proteins involved in regulating the translation and proliferation of these cells [48]. Jastrząb et al. demonstrated that the protective effect of CBD on UV-irradiated keratinocytes might be related to the increased activity of antioxidant enzymes such as superoxide dismutase and thioredoxin reductase. Moreover, this compound prevents lipid peroxidation by lowering the level of 4-HNE and 15d-PGJ2 and influences the interaction of NRF2-NFκB transcription factors [49]. The cytoprotective effect was also proven in the studies of Liu et al., who observed a decrease in the expression level of CASP1 and IL1B mRNA and decreased IL-1β after treatment with H_2_O_2_ keratinites. Moreover, the phytocannabinoid reduced pyroptosis induced by H_2_O_2_ and apoptosis [50]. Studies on keratinocytes and fibroblasts obtained from patients with psoriasis have shown that CBD can prevent structural and functional changes in the membranes of healthy skin cells arising during phototherapy, which is one of the methods of treating this disease [51]. According to scientific reports, the cytotoxicity of TER and other terpenes may result from increased cell membrane fluidity, interaction with the cell membrane, and modulation of the expression of genes closely related to apoptosis [52,53]. The cytotoxicity studies indicated that although the TER-loaded hydrogel is cytotoxic, loading both CBD and TER into the hydrogel structure does not cause the cytotoxic effect of the developed material. This study suggests that the created H-CBD and H-CBD-TER hydrogels may be safe DDSs with a wide range of therapeutic effects. However, further research, initially in vivo, is necessary to determine their actual effect on skin cells.

### 2.6. Assessment of Anti-Inflammatory Properties

**Figure 12:**
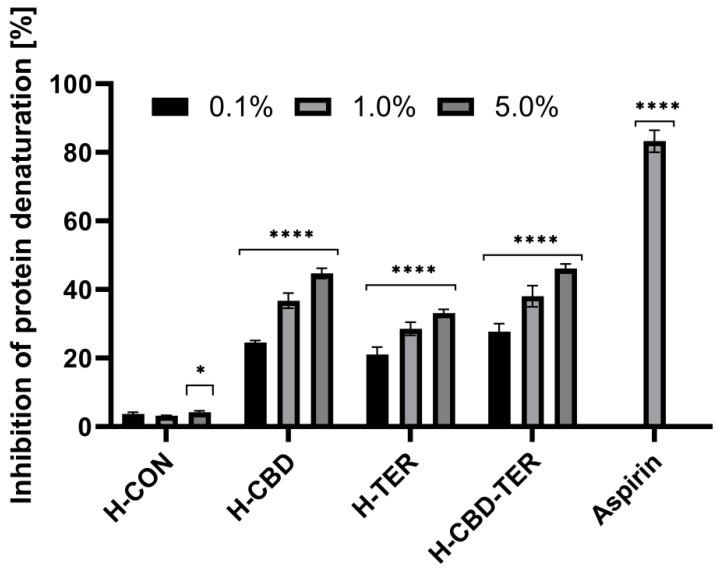
Inhibition of protein denaturation by tested hydrogels. **** *p* < 0.0001, * *p* = 0.0414 versus the control.

**Figure 13:**
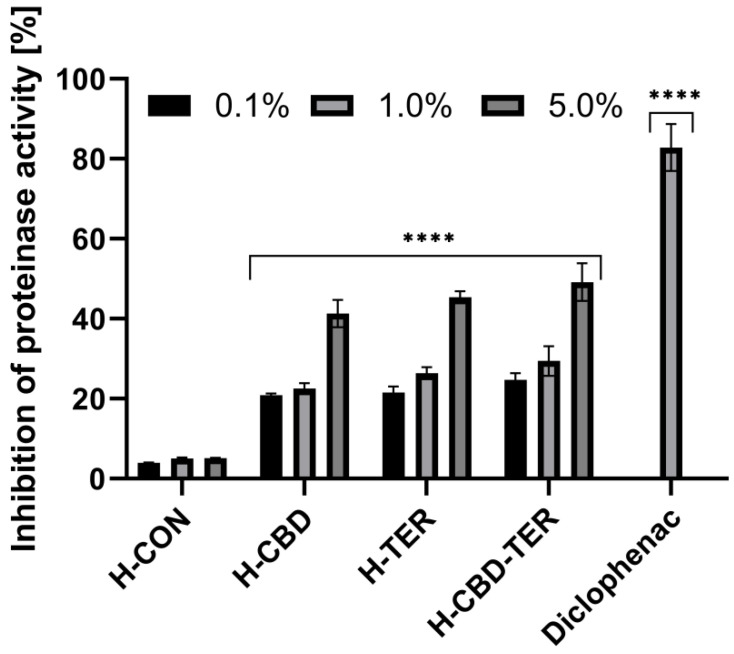
Inhibition of proteinase activity by tested hydrogels. **** *p* < 0.0001 versus the control.

**Figure 14:**
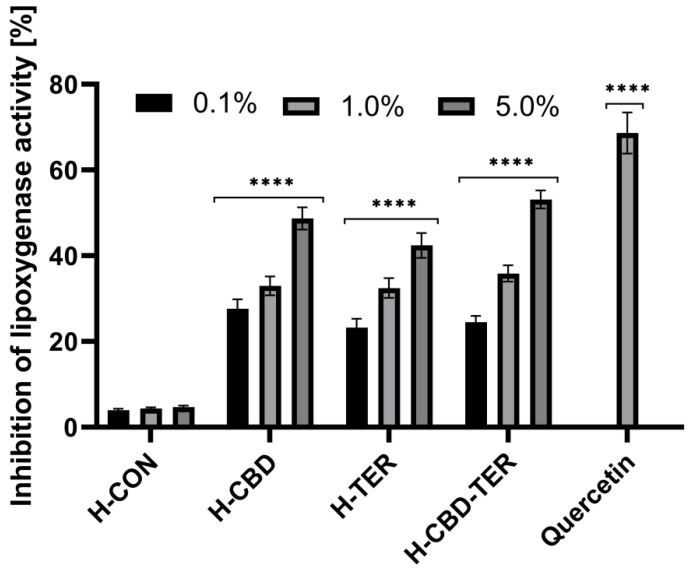
Inhibition of lipoxygenase activity by tested hydrogels. **** *p* < 0.0001 versus the control.

The anti-inflammatory properties of the developed hydrogels with CBD and TER were assessed using three in vitro assays. As part of these studies, the effect of the studied DDSs on protein (bovine serum albumin) denaturation and the activity of proteinase and lipoxygenase were assessed. The results showed that both CBD and TER hydrogels have anti-inflammatory activity. In contrast, the best properties were obtained when both compounds were loaded simultaneously into the structures of the developed hydrogel carriers. For this hydrogel, an inhibition of protein denaturation of more than 46.0% was achieved when the 5.0% hydrogel concentration was used (Figure 12). Similar results were observed in an evaluation of the inhibition of the activity of two tested enzymes, proteinase and lipoxygenase, for which the inhibition of activity in the case of 5.0% H-CBD-TER was noted by 49.0 and 53.0%, respectively. Hydrogels containing TER and CBD individually also showed anti-inflammatory action; however, the results of inhibition of denaturation and enzyme activity were slightly lower (Figure 13 and Figure 14).

Many skin conditions are related to inflammation [54]. As a result, developing DDSs containing active ingredients that control the pro-inflammatory response is extremely desirable. The results presented above illustrate the anti-inflammatory capabilities of CBD- and TER-containing hydrogels, which were confirmed in all three tests. Proteases are enzymes that can promote inflammation, mainly by regulating the expression and activity of various pro-inflammatory cytokines or chemokines. Hence, the ability of CBD and TER to inhibit proteinase activity may play an essential role in the anti-inflammatory activity of the developed DDSs [55]. In addition, the possibility of inhibiting lipoxygenase activity by the analyzed DDSs may contribute to reducing the production of pro-inflammatory mediators such as leukotrienes and affect the generation of anti-inflammatory mediators, which are lipoxins [56]. Tissue protein denaturation is a marker of inflammation and induces an inflammatory reaction. Hence, the possibility of inhibiting protein denaturation by the developed hydrogels shown in this study also confirms the anti-inflammatory properties of the studied biomaterials [57,58]. The anti-inflammatory effect of the studied DDSs, in addition to inhibiting the activity of pro-inflammatory enzymes and protein denaturation, is related to the multidirectional action of CBD, which can lower the level of pro-inflammatory cytokines, inhibit proliferation and induce apoptosis of T lymphocytes, and significantly reduce migration and adhesion of immune cells [16,59]. This phytocannabinoid can also inhibit the activation of pro-inflammatory cells, influence the synthesis of pro-inflammatory mediators, and reduce intracellular and mitochondrial oxidative stress [16,59]. CBD can also reduce reactive lipid peroxidation products and proteins, preventing changes in cell membranes’ physical properties and function [16].

### 2.7. Assessment of Antimicrobial Properties

The present study also assessed the inhibitory effect of the tested hydrogels on the growth of nine bacterial strains and two fungal strains. The results are given in Table 4. The tested hydrogels showed different abilities to inhibit the growth of the tested microorganisms. Most strains’ largest growth inhibition zones were observed for H-CBD-TER > H-TER > H-CBD > H-CON. The antibacterial effect was more substantial for the higher hydrogel concentration (5.0%). In the case of studies using bacterial strains, the effect of individual hydrogels depended on the strain, while in the case of fungal strains, hydrogels loaded with TER had stronger properties than those with CBD. No growth inhibition was noted for two of the tested strains, *Bacillus subtilis* and *Lactobacillus acidophilus*, in the case of any of the tested hydrogels. The analyzed hydrogels caused an inhibition zone from 5 to 36 mm in diameter, strictly dependent on the hydrogel’s type and the concentration of the microorganism used (Table 4).

In response to the constantly growing number of skin diseases caused by the excessive multiplication of microorganisms, both bacteria and fungi, on the skin surface, the possibility of inhibiting the growth of pathogenic microorganisms by active substances tested in this study gives an opportunity for the development of more effective therapeutic methods [60,61]. Other authors also described the antimicrobial properties of TER and proved the possibility of inhibiting the multiplication of many bacterial strains by this compound [25,62]. Although the mechanism of the antibacterial action of TER and other components of various essential oils has not been fully understood, many authors try to explain this action. Li et al. showed that this compound can induce various morphological changes in bacterial cells, reducing their size and causing an irregular cell shape. Also, microorganisms‘ cell walls and cell membranes may be disrupted, and the nucleus cytoplasm may be significantly reduced [62]. Moreover, literature data show that monoterpenes can expand bacterial cell membranes and increase their fluidity and permeability [63]. Additionally, they can lead to various disorders of the proper functioning of proteins embedded in the membrane, inhibit respiration, and negatively affect the ion transport process [26]. It is worth noting that the analyses carried out as part of this study showed that only the highest concentration of TER inhibits the growth of the probiotic bacteria *Lactobacillus acidophilus*, which positively affects the proper functioning of the skin [64].

Since many skin diseases are also associated with the development of strains of pathogenic fungi on the skin surface, the possibility of inhibiting the proliferation of *Candida albicans* and *Malassezia furfur* by the developed hydrogels containing CBD and TER indicates their great potential in the treatment of dermatological diseases. This antifungal effect may be related to the fact that, as research shows, TER may inhibit the metabolic pathways of fungi, and lead to hyphae distortion and disruption of spores [65]. TER may also cause disturbances of cell membrane integrity, alteration of extracellular conduction, loss of cellular components, and mycelial distortion [66].

The second active substance used in the developed hydrogels, CBD, also has documented antimicrobial properties, including bactericidal and bacteriostatic properties [21,67]. This compound can inhibit the growth of many Gram-positive and Gram-negative bacteria, including many strains showing drug resistance [21]. Blaskovich et al. additionally indicated that it also shows intense activity against the formation of bacterial biofilms, has local in vitro effectiveness, and has only a slight tendency to induce bacterial resistance. The primary mechanism responsible for the antibacterial properties of CBD is probably damage to the cell membrane of these microorganisms [21]. Kosgodage et al. showed that this compound is a potential inhibitor of the release of membrane vesicles from bacteria, which play an essential role in cell communication and interactions between the pathogen and the host, which affects the antibiotic resistance of these bacteria [68]. Moreover, these studies suggest that CBD may be a potential adjuvant in enhancing the activity of antibiotics used to combat a broad spectrum of bacteria [68].

As shown in this study, CBD may also have an inhibitory effect on the growth of fungi. This activity was also shown in the work of Feldman et al., who demonstrated that this compound can significantly inhibit biofilm formation by *C. albicans*, which significantly reduces the virulence of this microorganism. Additionally, this compound reduces the gene expression in exopolysaccharide production, hyphal formation, and virulence. Moreover, CBD reduces the intracellular ATP level, causes hyperpolarization of the mitochondrial membrane, changes the structure of the cell wall, and increases the permeability of the fungal cell membrane [67]. Thus, using hydrogels containing TER and CBD compounds and applying them directly to the skin can significantly minimize skin changes induced by pathogenic microorganisms’ activity.

### 2.8. Assessment of Anti-Aging Properties

**Figure 15:**
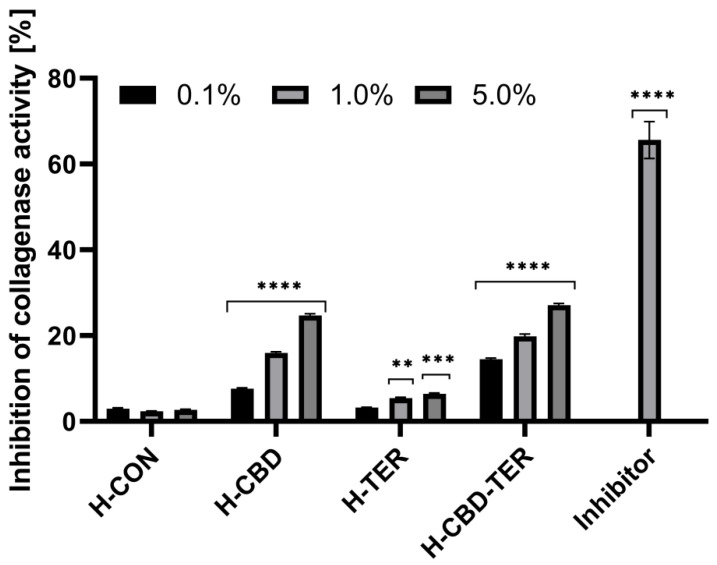
Inhibition of collagenase activity by the tested hydrogels. **** *p* < 0.0001, *** *p* = 0.0002, ** *p* = 0.0012 versus the control.

**Figure 16:**
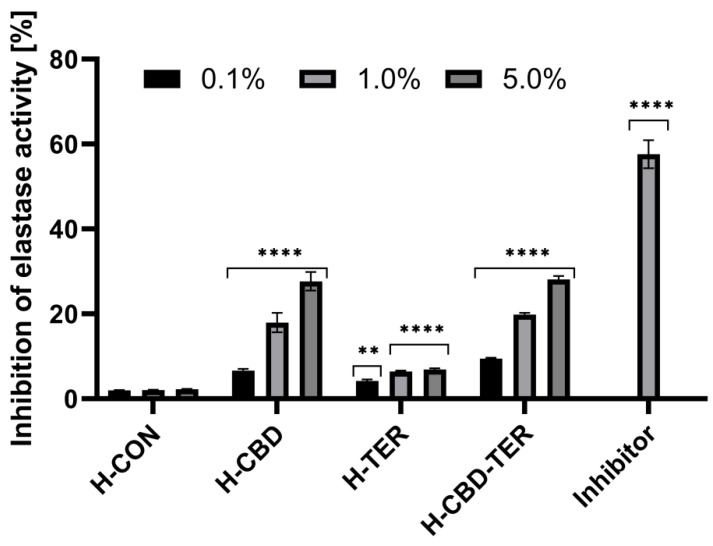
Inhibition of elastase activity by the tested hydrogels. **** *p* < 0.0001, ** *p* = 0.0095 versus the control.

In the subsequent study stage, the possibility of the tested hydrogels influencing the activity of two enzymes that degrade collagen and elastin fibers was investigated. For this reason, collagenase and elastase activities were determined. The results show that only hydrogels containing CBD, specifically H-CBD and H-CBD-TER, limit the activity of these enzymes. This impact is concentration-dependent, with the maximum concentration (5.0%) of H-CBD-TER inhibiting collagenase and elastase by 27.1 and 28.1%, respectively. H-CBD produced similar results, but H-TER had no significant effect on the activity of these enzymes (Figure 15 and Figure 16).

The ability to inhibit the activity of collagenase and elastase is an essential element in assessing the anti-aging properties of active compounds, both synthetic and of natural origin [69]. These enzymes are responsible for the breakdown of elastin and collagen in the extracellular matrix, which significantly affects the loss of strength and elasticity of the skin and interferes with proper wound healing and skin regeneration [70,71]. Since their activity can be induced by various external factors, such as free radicals, UV radiation, and genetic conditions, compounds that can reduce the activity of these enzymes are constantly searched for [72]. In previous research, we showed that Cannabis sativa herb extracts can decrease the activity of these enzymes, which is most likely the result of the interaction of physiologically active chemicals found in this extract [71]. This work shows the possibility of inhibiting these two matrix metalloproteinases by CBD-loaded hydrogel and carriers containing both CBD and TER. Since the endocannabinoid system (ECS) receptors, CB1 and CB2, have endogenous ligands located in the skin, cannabinoid compounds, including CBD, can act as a stimulant or an inhibitory agent for the ECS, responsible for maintaining homeostasis, regulating sebum production, inhibiting or promoting the proliferation of keratinocytes, or inhibiting inflammatory promoters [73,74,75]. Thus, the regenerating and protective effects of cannabinoids and their positive effect on the skin aging processes may result from both the influence on the ECS and the inhibition of the activity of collagenase and elastase. As a result, innovative drug carriers containing molecules that can slow the aging process and increase skin regeneration are crucial in modern dermatology and cosmetology.

## 3. Materials and Methods

### 3.1. Materials

Acetic acid (CH₃COOH, ≥99.0%, Sigma-Aldrich, Poznan, Poland), ε-Caprolactone (2-Oxepanone, CL, 99.0%, Aldrich, Poznan, Poland), chitosan (CHT, low molecular weight, 75.0% deacetylated, Sigma-Aldrich, Poznan, Poland), diethylene glycol (DEG, ≥99.8%, Sigma-Aldrich, Poznan, Poland), dibutyltin dilaurate (DBDLSn, >96.0%, Sigma-Aldrich, Poznan, Poland), dichloromethane (DCM, CH_2_Cl_2_, ≥99.8%, POCh, Gliwice, Poland), 1,6-diisocyanatohexane (hexamethylene diisocyanate, HDI, 98.0%, Aldrich, Poznan, Poland), poly(ethylene glycol) (PEG, *M_n_* = 1000 g/mol, pure, Sigma-Aldrich, Poznan, Poland), poly(tetrahydrofuran) (PTHF) (Sigma-Aldrich, Poznan, Poland, *M_n_* = 2000 g/mol), methanol (MeOH, Chempur, Warsaw, Poland), immobilized lipase B from *Candida Antarctica* (CA) (Sigma-Aldrich, Poznan, Poland), N,N-dimethylformamide (DMF, anhydrous, 99.8%, Sigma-Aldrich, Poznan, Poland), acetone (ACS reagent, ≥99.5%, Sigma-Aldrich, Poznan, Poland), phosphate buffer solution (pH 7.40 ± 0.05, 0.1 M, PBS, potassium dihydrogen phosphate/di-sodium hydrogen phosphate, 20.0 °C, Avantor Performance Materials, Gliwice, Poland), and potassium acetate buffer solution (100 mM, pH 5.5, 0.2 μM filtered, Avantor Performance Materials, Gliwice, Poland) were used as received.

### 3.2. Enzyme Ring-Opening Polymerization of ɛ-Caprolactone and Poly(ethylene glycol)

The enzyme ring-opening polymerization (e-ROP) reactions of CL and PEG were carried out according to our previously described method with some modifications [76,77,78]. Before the reaction, CL, PEG, and CA were dried under a vacuum at room temperature for 1 h. Next, 0.05 mol CL was placed in a three-neck flask equipped with a stirrer and thermometer (under an argon atmosphere), and 20 mL of toluene was added. The mixture was stirred at 80.0 °C for 4 h. Next, an appropriate amount of PEG and CA (500 mg) was added to the mixture. Stirring was continued at 80.0 °C for 72 h under an argon atmosphere. After this time, the enzyme was filtered off. Toluene was removed by evaporation under reduced pressure at room temperature. Next, the cooled product was dissolved in DCM and extracted with cold methanol and distilled water.

The structures of the synthesized copolymers were evaluated using ^1^H and ^13^C NMR techniques (Figures S1 and S2, Supplementary Materials). The spectra were recorded on an Agilent Technologies 400 MHz (Santa Clara, CA, USA) spectrometer. The IR spectra were measured from KBr pellets (PerkinElmer spectrometer, Great Britain) (Figure S3, Supplementary Materials).

Spectroscopy data of obtained copolymers:

The ^1^H NMR (CDCl_3_): 1.40 ppm (-CO-CH_2_-CH_2_-CH_2_-CH_2_-CH_2_-O-), 1.64 ppm (-CO-CH_2_-CH_2_-CH_2_-CH_2_-CH_2_-O-), 2.30 ppm (-CO-CH_2_-CH_2_-CH_2_-CH_2_-CH_2_-O-), 3.63 ppm (-CH_2_-CH_2_-O-), 4.05 ppm (-CO-CH_2_-CH_2_-CH_2_-CH_2_-CH_2_-O-), 4.21 ppm (-CH_2_-CH_2_-O-CH_2_-CH_2_-O-Cap-); the ^13^C NMR (CDCl_3_): 24.62 ppm (-CO-CH_2_-CH_2_-CH_2_-CH_2_-CH_2_-O-), 25.57 ppm (-CO-CH_2_-CH_2_-CH_2_-CH_2_-CH_2_-O-), 28.39 ppm (-CO-CH_2_-CH_2_-CH_2_-CH_2_-CH_2_-O-), 32.36 ppm (-CO-CH_2_-CH_2_-CH_2_-CH_2_-CH_2_-OH) end groups, 34.16 ppm (-CO-CH_2_-CH_2_-CH_2_-CH_2_-CH_2_-O-), 62.57 ppm (-CO-CH_2_-CH_2_-CH_2_-CH_2_-CH_2_-OH) end groups, 63.49 ppm (-CH_2_-CH_2_-O-CH_2_-CH_2_-O-CO-), 64.18 ppm (-CO-CH_2_-CH_2_-CH_2_-CH_2_-CH_2_-O-), (-CH_2_-CH_2_-O-CH_2_-CH_2_-O-CO-), 70.60 ppm (-CH_2_-CH_2_-O-), 173.57 ppm (-CO-CH_2_-CH_2_-CH_2_-CH_2_-CH_2_-O-).

FTIR (KBr, cm^−1^): 2941 (ν_as_CH_2_), 1722 (νC=O), 1236 (νC-O).

The *M*_n_ and *Đ* index values of the synthesized copolymers were determined using the GPC technique. The measurements were carried out on a Malvern Viscotek GPCMax TDA 305 (Malvern Panalytical, Malvern, UK) chromatograph equipped with a Jordi Gel DVB mixed-bed column (Jordi Labs, Mansfield, MA, USA). The mobile phase flow (DCM) was set to 1.0 mL/min, and the column temperature was set to 30 °C. The system was calibrated using polystyrene standards.

### 3.3. Hydrogels’ Preparation and Characterization

Hydrogels were obtained using a previously developed and appropriately modified three-step method [11,79]. In the first step, CL and PEG copolymers were synthesized. The prepolymers of HDI and CL-PEG copolymer, PTHF, and DEG were obtained in the second step. Next, in the final step, hydrogels were prepared from CHT, HDI, DEG, PTHF, and CL-PEG.

The prepolymers were obtained through a polyaddition reaction between HDI, CL-PEG copolymer, PTHF, and DEG in a molar ratio of 2.1:0.7:0.2:0.1 using 3 drops of 0.1 wt % DBDLSn solution in toluene as a catalyst. The reactions were performed at 80.0 °C for 3 h under an argon atmosphere to form an isocyanate-terminated prepolymer. Next, CHT was dispersed into a glacial acetic acid/DMF mixture (30 mL) in a ratio of 50/50 and left overnight to obtain good swollen CHT. Next, the obtained prepolymer was added to the dispersion of CHT. The reactions were carried out in an NCO (prepolymer)/OH (or NH_2_) (CHT) molar ratio of 1.65:1 at 80.0 °C for 4 h under an argon atmosphere. The reaction mixture was then transferred to the distilled water. The precipitated products were separated by filtration and washed with DMF, methanol, and acetone. The final products were dried under a vacuum for two weeks at room temperature.

The mass swelling ratio (*MSR*) of obtained hydrogels was determined at 37.0 °C during incubation in a buffer. Samples in triplicate were submerged in a buffer solution (20 mL) for a given time, and their weights were taken after removing the excessive surface water. The mass swelling ratio was calculated using the following formula:

*MSR* = ((W_2_ − W_1_)/W_1_)/100% (1)

where the following definitions hold:

W_1_ is the weight of the initial hydrogel;
W_2_ is the weight of the swollen hydrogel.

In order to evaluate the percentage of degradation, the hydrogel samples were immersed in buffer at 37.0 °C for 8 weeks; most importantly, the medium was replaced with fresh buffer every week. At the end of the experiment, the samples were dried in a vacuum for 48 h. The degree of degradation of hydrogels (in triplicate) was determined by the weight loss (*WL*) of the samples according to the following equation:

*WL* = [(W_1_ − W_2_)/W_1_]/100% (2)

where the following definitions hold:

W_1_ is the weight of the dry sample before degradation;
W_2_ is the weight of the dry sample after degradation.

### 3.4. In Vitro Release Studies of Active Substances from Hydrogels

CBD and/or TER were/was loaded into the hydrogel by physical mixing using the following procedure: A total of 5.0% (m/m) of CBD and TER in distilled water/Tween 80 mixture (2.0% (w/v) was added to three hydrogel samples (H-TER, H-CBD-TER, H-CBD). The hydrogels were left sealed for 24 h. The mixtures were dried under vacuum at room temperature to obtain an active-substance-loaded hydrogel film. The in vitro release of CBD or TER from the hydrogels was performed in a buffer (pH 7.4 or 5.5) containing 2.0% (*w*/*v*) Tween 80 at 37 °C under stirring. Vials containing hydrogel films were filled with 5.0 mL of a buffer, sealed, and left at 37.0 °C for a specified time. The solutions were then removed for further testing and replaced by fresh buffer.

The amount of (cannabidiol) CBD being released was measured by UV-Vis spectroscopy (detected at the wavelength of 220 and 280 nm) [80,81].

The amount of (α-terpineol) TER being released was measured by UV-Vis spectroscopy (detected at the wavelength of 205 nm due to the π–π* transition of the double bond in TER) [82].

The release data points were subjected to zero-order kinetics, first-order kinetics, and Korsmeyer–Peppas models. Calculations were made based on the formulas mentioned below [36].

(3) Zero-order: F=kt

(4) $$\mathrm{First}\text{-}\mathrm{order}:\;\log F=\log F_{0}-\frac{kt}{2.303}$$

(5) $$\mathrm{Korsmeyer}–\mathrm{Peppas}\;\mathrm{model}:\;F=kt^{n}(F<0.6)$$

where the following definitions hold:

F is the fraction of the drug released from the matrix after time;
$F_{0}$ is the initial amount of the drug;
k is a model constant, and *n* is the drug release exponent in the Korsmeyer–Peppas model.

### 3.5. Antioxidant Properties

#### 3.5.1. DPPH Radical Scavenging Assay

To evaluate the antioxidant properties of H-CON, H-CBD, H-TER, and H-CBD-TER hydrogels, a 1,1-diphenyl-2-picrylhydrazyl (DPPH) radical scavenging test was performed according to the methodology described by Brand-Williams et al. [83] with minor modifications. The test was performed on 96-well plates, where 50 µL of individual dilutions of the analyzed hydrogels (in the concentration range from 0.1–5.0%) in distilled water was added to each well. Then, 150 µL of DPPH working solution in methanol (4 mM) was added to the wells. The samples were mixed thoroughly, and the absorbance at λ = 516 nm was measured with a FilterMax F5 microplate reader (Thermo Fisher Scientific, Waltham, MA, USA). Measurements were taken every 5 min for 30 min. All samples were analyzed in triplicate. Distilled water mixed with a DPPH working solution was used as a control. Measurements were performed in triplicate for each test sample. The DPPH radical scavenging activity was calculated using the following equation:

(6) $$\%\;DPPH\;scavenging=\frac{Ac-As}{Ac}\times 100\%$$

where the following definitions hold:

Ac is the absorbance of the control sample;
As is the absorbance of the test sample.

#### 3.5.2. ABTS Radical Scavenging Assay

The second test to evaluate the antioxidant activity of the tested hydrogels was the ABTS radical (2,2’-azino-bis (3-ethylbenzothiazoline-6-sulfonic acid) diammonium salt) scavenging assay based on the procedure described by Ziemlewska et al. [84]. Initially, 19.5 mg of ABTS and 3.3 mg of potassium persulfate were mixed with 7 mL phosphate buffer (pH = 7.4). The mixture prepared this way was dissolved by stirring for 16 h in the dark. The solution was then diluted until an absorbance of approximately 1.0 was obtained (measured at λ = 414 nm). In the next step, individual dilutions of the test samples were mixed with 980 µL of diluted ABTS solution and incubated for 10 min in the dark. Finally, the decrease in ABTS absorbance at λ = 734 nm was measured using a DR600 UV-Vis spectrophotometer (Hach Lange, Wrocław, Poland). Distilled water was used as a blank. The measurements were carried out in triplicate for each concentration of hydrogels with CBD, TER, a combination of these compounds, and the hydrogel matrix without adding these bioactive compounds. The ABTS scavenging activity was calculated from the following equation:

(7) $$\%\;of\;ABTS\;radical\;scavenging=1-\frac{As}{Ac}\times 100\%$$

#### 3.5.3. Detection of Intracellular Levels of Reactive Oxygen Species (ROS)

In order to determine the ability of the analyzed hydrogels (H-CON, H-CBD, H-TER, and H-CBD-TER) to inhibit the intracellular production of reactive oxygen species in keratinocytes (HaCaT) and fibroblasts (BJ), the fluorogenic H_2_DCFDA dye was used based on the procedure described by Nizioł-Łukaszewska et al. [85]. Briefly, cells were seeded in 96-well flat-bottom plates at a density of 2 × 10^4^ cells per well. The cells were then cultured in an incubator for 24 h to adhere them to the bottom of the wells. The DMEM medium was then removed and replaced with 10 µM H_2_DCFDA indicator solution (Sigma Aldrich, Sant Louis, MO, USA) dissolved in serum-free DMEM medium (FBS). HaCaT and BJ cells were incubated with H_2_DCFDA for 45 min and then treated with H-CON, H-CBD, H-TER, and H-CBD-TER hydrogels in 0.1–5.0% concentration range. Cells treated with 1 mM hydrogen peroxide (H_2_O_2_) solution were the positive control, while cells untreated with analyzed hydrogels were the control sample. Fluorescence of 2′,7′-dichlorofluorescein (DCF) in individual wells was measured every 30 min for 90 min at excitation wavelength λ = 485 nm and emission λ = 530 nm using a microplate reader (FilterMax F5, Thermo Fisher Scientific, Waltham, MA, USA). Three independent experiments were performed, and each sample was tested in three replications.

#### 3.5.4. Determination of Superoxide Dismutase Activity (SOD)

The antioxidant properties of the tested hydrogels (H-CON, H-CBD, H-TER, and H-CBD-TER) were also assessed by analyzing their influence on the activity of the antioxidant enzyme superoxide dismutase (SOD), which protects cells against reactive oxygen species. For this purpose, the Colorimetric Superoxide Dismutase Activity Assay kit (ab65354, Abcam, Cambridge, UK) was used. The analyses were performed for all types of hydrogels at concentrations of 0.1, 1.0, and 5.0%. According to the recommendations of the kit manufacturer, three blank samples were prepared. For this purpose, 20 µL of ddH_2_O (blank 1), 20 µL of test samples (blank 2), and 20 µL of ddH_2_O (blank 3) were added to the wells. Then, 200 µL of WST working solution was added to all wells. In the next step, 20 µL of SOD dilution buffer was added to blank 2 and blank 3. Then, 20 µL of enzyme working solution was added to all wells with test samples and blank 1. The samples prepared this way were mixed and incubated at 37 °C for 20 min. The absorbance of the analyzed samples was then measured at λ = 450 nm with a microplate reader (FilterMax F5, Thermo Fisher Scientific, Waltham, MA, USA). Three independent experiments were carried out in which each sample was prepared in duplicate. The ability to inhibit SOD activity by the analyzed hydrogel samples was calculated using the following equation:

(8) $$\%\;SOD\;activity=\frac{\left(A\;blank\;1-A\;blank\;3\right)-(A\;sample-A\;blank\;2)}{\left(A\;blank\;1-A\;blank\;3\right)}\times 100\%$$

### 3.6. Cytotoxicity Assays

#### 3.6.1. Cell Culture and Preparation of Hydrogel Extracts

Cytotoxicity analyses of the analyzed hydrogels were performed on normal human HaCaT keratinocytes (CLS Cell Lines Service GmbH, Eppelheim, Germany) and BJ fibroblasts (American Type Culture Collection, Manassas, VA 20108, USA). Cells were grown in culture flasks in an incubator at 37.0 °C in a humidified atmosphere of 95.0% air and 5.0% carbon dioxide. Cells were grown in Dulbecco’s Modified Eagle Medium (DMEM, Biological Industries, Cromwell, CO, USA) supplemented with sodium pyruvate and L-glutamine with high glucose (4.5 g/L). The medium was additionally supplemented with 10.0% fetal bovine serum (Gibco, Waltham, MA, USA) and 1.0% antibiotics (100 U/mL penicillin and 1000 µg/mL streptomycin, Gibco) to prevent contamination of the culture medium. Cells were passaged when a confluence of approximately 60–80.0% was achieved. Extracts of the test hydrogels were prepared by shaking the hydrogels in the DMEM culture medium for 24 h. Hydrogel concentrations of 0.01, 0.1, 0.5, 1.0, 2.5, and 5.0% were prepared for all hydrogels (H-CON, H-CBD, H-TER, and H-CBD-TER). Prepared dilutions of hydrogels were sterilized using membrane filters (0.22 µm).

#### 3.6.2. Alamar Blue Assay

The first test used to assess the cytotoxicity of the tested hydrogels was a test using a resazurin-based solution (Sigma, R7017, Life Technologies, Bleiswijk, The Netherlands), which allows the measurement of the reduction power of cells. In the first step, fibroblasts and keratinocytes were seeded separately in 96-well sterile flat-bottom plates (VWR, Radnor, PE, USA). After 24 h, the cultured cells were attached to the bottom of the plates, and the cells were exposed to test hydrogels in a concentration range of 0.01–5.0% for 24 h. Then, hydrogel solutions were aspirated, and 60 µM resazurin solution was added to the wells on a microplate. The prepared plates were incubated at 37.0 °C for 2 h. The fluorescence of the hydrogel-treated cells was then measured at λ = 570 nm using a microplate reader (Thermo Fisher Scientific, Waltham, MA, USA). The control sample consisted of cells (HaCaT and BJ separately) grown in a DMEM medium without adding hydrogels (100% viability). Three independent experiments were performed for each hydrogel concentration test in triplicate.

#### 3.6.3. Neutral Red Uptake Assay

Another test used to assess the viability of HaCaT and BJ cells exposed to the tested hydrogels was the Neutral Red Uptake Capture Assay (Sigma Aldrich, Poznań, Poland) based on the previously described procedure [11]. Cells were exposed to solutions of tested hydrogel extracts (H-CON, H-CBD, H-TER, and H-CBD-TER) at concentrations of 0.01–5.0% for 24 h. After this time, cells were treated with neutral red dye (40 µg/mL) for 2 h. The cells were washed twice with phosphate-buffered saline (PBS) and treated with a decolorizing buffer (C_2_H_5_OH/CH_3_COOH/H_2_O, 50.0%/1.0%/49.0%). After the cells were shaken on an orbital shaker for 15 min, the uptake of neutral red dye was determined by measuring the absorbance of the samples at λ = 540 nm using a microplate reader (Thermo Fisher Scientific, Waltham, MA, USA). The control sample consisted of cells (separately HaCaT and BJ) maintained in a DMEM medium without adding hydrogel extracts, for which the viability was assumed to be 100%. As part of the analyses, three independent experiments were carried out for which each concentration of hydrogel extracts was assessed in three replications.

### 3.7. Determination of Anti-Inflammatory Properties

#### 3.7.1. Assessment of Inhibition of Protein Denaturation

The anti-inflammatory properties of the tested hydrogels with CBD and TER were assessed using the methodology described by Sarvesvaran et al. [86]. As part of this test, the possibility of inhibiting the denaturation of bovine serum albumin (BSA) by hydrogels in the concentration range of 0.1, 1.0, and 5.0% was assessed. Briefly, 1000 µL of individual concentrations of hydrogel extracts (H-CON, H-CBD, H-TER, and H-CBD-TER) was mixed with 450 µL of 5.0% aqueous BSA solution and 1400 µL of phosphate-buffered saline (PBS, pH 6.4). The mixtures prepared this way were incubated at 37.0 °C for 15 min. The samples were then heated at 70.0 °C for 5 min, followed by cooling in an ice bath to 25.0 °C. Then, the absorbance of the tested samples was measured at λ = 660 nm using the DR600 UV-Vis spectrophotometer (Hach Lange, Wrocław, Poland). A diclofenac concentration of 500 µg/mL was used as a positive control. Three independent experiments were performed as part of the analyses, and each sample was tested in triplicate. The inhibition of protein denaturation by the tested hydrogels was calculated from the following equation:

(9) $$\%\;inhibition\;of\;denaturation=\frac{1-As}{Ac}\times 100\%$$

where the following definitions hold:

As is the absorbance of the tested sample;
Ac is the absorbance of the control sample.

#### 3.7.2. Assessment of Inhibition of Lipoxygenase Activity

The anti-inflammatory activity of the analyzed hydrogels was verified by assessing the possibility of inhibiting the activity of the lipoxygenase enzyme according to the methodology described by Ziemlewska et al. [84]. Initially, 10 µL of hydrogel extracts was tested (at 0.1, 1.0, and 5.0% concentrations) with 160 µL of 100 mM PBS and 20 µL of soybean lipoxygenase solution (167 U/mL). The samples were then incubated at 25.0 °C for 10 min. Sequentially, 10 µL of linoleic sodium was added to each well to initiate the enzymatic reaction. The absorbance of the analyzed samples was measured every minute for 3 min at λ = 234 nm using a microplate reader (Thermo Fisher Scientific, Waltham, MA, USA). Diclofenac was used as a standard inhibitor at a concentration of 500 µg/mL. As part of the work, three independent experiments were performed in which all concentrations of hydrogel extracts were tested in three replications. The percentage of inhibition of lipoxygenase activity was calculated from the following equation:

(10) $$\%\;inhibition\;of\;lipoxygenase\;activity=\frac{Ac-As}{Ac}\times 100\%$$

where the following definitions hold:

As is the absorbance of the tested sample;
Ac is the absorbance of the control sample.

#### 3.7.3. Assessment of Inhibition of Proteinase Activity

Another test to evaluate the anti-inflammatory properties of the analyzed hydrogels was the proteinase inhibition test carried out according to the method described by Juvekar et al. [80], with minor modifications by Gunathilake et al. [87]. A 1.0% trypsin solution dissolved in 20 mM Tris-HCl buffer (pH 7.4) was mixed with the hydrogel extract samples (at concentrations 0.1, 1.0, and 5.0%). The solutions prepared this way were incubated at 37.0 °C for 5 min. In the next step, 0.8% (*w*/*v*) casein was added to the solutions and incubated for another 20 min. Then, 70.0% perchloric acid was added to stop the enzymatic reaction. The samples were then centrifuged, and the absorbance at λ = 210 nm was measured using a DR600 UV-Vis spectrophotometer (Hach Lange, Wrocław, Poland). The reaction buffer was used as a blank. A phosphate buffer solution was used as a control. Three independent experiments were carried out in which all samples were tested in triplicate. The percent inhibition of protein denaturation was calculated according to the following equation:

(11) $$\%\;inhibition\;of\;proteinase\;activity=100\times \frac{1-A2}{A1}$$

where the following definitions hold:

A1 is the absorbance of the control sample;
A2 is the absorbance of the test sample.

### 3.8. Assessment of Anti-Aging Properties

#### 3.8.1. Determination of Anti-Elastase Activity

The evaluation of the anti-aging properties of CBD- and TER-loaded hydrogels was performed using a fluorimetric kit (Abcam, ab118971, Cambridge, MA, USA), assessing the possibility of inhibiting the activity of the enzyme neutrophilic elastase (NE). The analyses were performed based on the instructions provided by the manufacturer according to the procedure previously described by Zagórska-Dziok et al. [88]. Fluorometric measurements of elastase activity were performed in a 96-well plate with black walls and a clear bottom. As part of the analysis, analyses were carried out for extracts from all four tested hydrogels at concentrations of 0.1, 1.0, and 5.0%. Initially, NE enzyme solutions, NE substrate, and inhibitor control (succinyl-alanyl-alanyl-prolyl-valine chloromethyl ketone; SPCK) were prepared according to the manufacturer’s instructions. The NE solution was then added to all reaction wells. Next, test solutions of hydrogel extracts, inhibitor control, and enzyme control (assay buffer) were added to each well and mixed in the dark at 37.0 °C for 5 min. Meanwhile, a reaction mixture was prepared by mixing an assay buffer and NE substrate. The prepared solution was added to each well, and the fluorescence was immediately measured at excitation wavelength λ = 400 nm and emission λ = 505 nm using a microplate reader (FilterMax F5, Thermo Fisher Scientific, Waltham, MA, USA). Measurements were performed in the kinetic mode (for 30 min at 37.0 °C). Two independent experiments were performed in which all samples were analyzed in duplicate. The influence of the tested hydrogels on NE activity was calculated using the following equation:

(12) $$\%\;relative\;activity=\frac{∆RFU\;test\;inhibitor}{∆RFU\;enzyme\;control}\times 100\%$$

#### 3.8.2. Determination of Anti-Collagenase Activity

The study also assessed H-CON, H-CBD, H-TER, and H-CBD-TER hydrogels in terms of their ability to inhibit collagenase activity, which is responsible for the hydrolysis of collagen fibers. For this purpose, a fluorimetric kit (Abcam, ab211108, Cambridge, MA, USA) was used, and the determinations were carried out according to the manufacturer’s instructions and the procedure previously described by Nowak et al. [89]. Collagenase inhibition activity was assessed in a black 96-well plate with a clear flat bottom for all four tested hydrogels at concentrations of 0.1, 1.0, and 5.0%. Collagenase (COL) was first dissolved in a collagenase analysis buffer (CAB) and added to all hydrogel samples tested. The inhibitor control solution consisted of a collagenase inhibitor (1,10-phenanthroline (80 mM)), a collagenase enzyme, and a CAB buffer. An enzyme control was prepared by mixing diluted COL with CAB. CAB buffer was used as background control. The prepared samples were shaken gently on an orbital shaker for 15 min at room temperature in the dark. The reaction mixture was then prepared by mixing the collagenase substrate with CAB buffer and added to all analyzed samples. Finally, the fluorescence was measured at excitation wavelength λ = 490 nm and emission at λ = 520 nm with a microplate reader (FilterMax F5, Thermo FisherScientific, Waltham, MA, USA). Measurements were carried out in the kinetic mode at 37.0 °C for 60 min. Two independent experiments were performed in which individual dilutions of hydrogels were prepared in duplicate. The ability to inhibit the activity of COL by the test samples was calculated from the following equation:

(13) $$\%\;relative\;COL\;inhibition=\frac{enzyme\;control-sample}{enzyme\;control}\times 100\%$$

### 3.9. Assessment of Antimicrobial Activity

The disc diffusion method was used to assess the effect of the analyzed hydrogels on the growth of microorganisms (pathogenic and probiotic bacteria or fungi). The analyzed strains were obtained from the American Type Culture Collection (Manassas, USA). The organisms that were used in the tests were bacteria *Staphylococcus aureus* ATCC^®^ BAA-2312™, *Staphylococcus epidermidis* ATCC^®^ 49134™, *Staphylococcus capitis* ATCC^®^ 146™, *Micrococcus luteus* ATCC^®^ 10240™, *Propionibacterium acnes* ATCC^®^ 11827™, *Corynebacterium* ATCC^®^ 373™ ATCC^®^ 11827™, *Bacillus subtilis* ATCC^®^ 11774™, and *Kocuria kristinae* ATCC^®^ BAA-752™ and the probiotic *strain Lactobacillus acidophilus* ATCC^®^ 314™. Additionally, determination of the zone of inhibition of growth was performed for *Candida albicans* ATCC^®^ 14053™ and *Malassezia furfur* ATCC^®^ 14521™ fungi. Initially, 10 mL samples of agar media appropriate for each bacterial and fungal strain were poured onto sterile Petri dishes. For this purpose, Tryptic Soy Agar, Mannitol Salt Agar, Nutrient Agar, Tryptone Soya Agar Blood Agar, Blood Agar, LB Agar, Brain Heart Infusion Agar, Yeast Malt Agar, Sabouraud Dextrose Agar, and MRS Agar were used. Then, the media were inoculated with individual strains of microorganisms with a density of 5.0 × 10^7^ CFU/mL. Then, extracts of the analyzed hydrogels were prepared at 1.0% and 5.0% concentrations and sterilized by membrane filters (0.22 µm). Sterile discs of filter paper (6 mm in diameter) were soaked in various concentrations of hydrogels. A disc soaked in sterile distilled water was used as a negative control. The inoculated plates with the impregnated discs were left at 4.0 °C for 2 h to allow the compounds to pre-diffuse through the medium, and then the plates were transferred to an incubator. Cultures were carried out in aerobic or anaerobic conditions depending on the requirements of the microorganism. The effect of the test compounds on the growth of the tested microbial strains was assessed by measuring the diameter of the inhibition zone after 48 h of cultivation at 35.0 ± 2 °C.

### 3.10. Statistical Analysis

The values of individual measured parameters were expressed as mean ± standard deviation (SD). Two-way analysis of variance (ANOVA) and Bonferroni post-test were performed between groups at *p* < 0.05 to assess significant differences between the individual values. Statistical analyses were performed using GraphPad Prism 9.3.1 (GraphPad Software, Inc., San Diego, CA, USA).

## 4. Conclusions

Recently, there has been a growing interest in the use of phytocannabinoids, such as CBD, to treat various skin diseases. As a result, suitable carriers for this exceedingly versatile chemical which might be used in dermatology and cosmetology are required. It is also critical to release this substance in a controlled manner, not just on the skin’s surface but also in its deeper layers. In the present work, new biodegradable hydrogels derived from CL and PEG copolymers, DEG, PTHF, HDI, and CHT for controlled release of CBD and also TER have been obtained and characterized. It was found that both active substances were released from the developed hydrogels with relatively high control and rather near-first-order kinetics. Importantly, the “burst release” of CBD or TER has not been observed. The proposed combination of CBD with TER, a commonly known compound that increases skin penetration, may be a way to increase the effectiveness of CBD and significantly improve the treatment results. The beneficial features of the hydrogels presented in this work, such as antioxidant, anti-inflammatory, anti-aging, and antibacterial activity, highlight the validity of further research, including in vivo, to evaluate the properties and possible use of the developed hydrogel carriers.

## Figures and Tables

**Table 1:** Synthesis of CL-PEG copolymers.

Sample	Molar Ratio ^a^	Yield [%]	*M_n_*^b^ [g/mol]	*Đ* ^b^
CL-PEG A	30:1	92	2400	1.89
CL-PEG B	40:1	83	2700	1.93
CL-PEG C	50:1	74	3400	1.69
CL-PEG D	60:1	71	3600	2.29

^a^—CL/PEG molar ratio; PEG (*M_n_* = 1000 g/mol); ^b^—number average molar mass (*M_n_*) and dispersity index (*Đ*) determined by the GPC method; reaction conditions: temp. 80.0 °C; time—7 days; medium—toluene, CA (500 mg).

**Table 2:** Analysis of CBD and TER release results from obtained hydrogels using mathematical models.

No.	Zero Order Model	First Order Model	Korsmeyer–Peppas Model		Transport Mechanism
	*R* ^2^	*R* ^2^	*R* ^2^	*n*	
H-CBD ^a^	0.927	0.947	0.827	0.503	non-Fickian transport
H-TER ^a^	0.883	0.932	0.858	0.412	Fickian transport
CBD (H-CBD-TER) ^a^	0.931	0.972	0.845	0.511	non-Fickian transport
TER (H-CBD-TER) ^a^	0.915	0.859	0.780	0.435	Fickian transport
H-CBD ^b^	0.905	0.918	0.775	0.383	Fickian transport
H-TER ^b^	0.770	0.971	0.793	0.292	Fickian transport
CBD (H-CBD-TER) ^b^	0.910	0.933	0.771	0.404	Fickian transport
TER (H-CBD-TER) ^b^	0.832	0.897	0.788	0.317	Fickian transport

^a^ pH 7.4, ^b^ pH 5.5.

**Table 3:** Antioxidant properties of the tested hydrogels.

	Scavenging the DPPH Radical [%]						Scavenging the ABTS Radical [%]					
	0.01%	0.1%	0.5%	1.0%	2.5%	5.0%	0.01%	0.1%	0.5%	1.0%	2.5%	5.0%
H-CON	3.12 ± 0.5	3.33 ± 0.5	4.56 ± 0.3	4.02 ± 0.2	5.33 ± 0.2	6.04 ± 0.4	3.5 ± 0.3	3.42 ± 0.4	5.33 ± 0.5	5.65 ± 0.5	5.47 ± 0.5	6.93 ± 0.5
H-CBD	15.22 ± 1.5	35.77 ± 3.5	56.34 ± 6.2	67.33 ± 4.4	78.33 ± 5.9	83.21 ± 9.1	46.55 ± 1.5	67.3 ± 3.5	74.67 ± 6.3	86.7 ± 7.7	89.55 ± 8.8	91.43 ± 6.1
H-TER	1.23 ± 0.12	1.77 ± 0.3	2.77 ± 0.2	2.19 ± 0.2	3.67 ± 0.4	6.77 ± 0.3	3.44 ± 0.5	4.44 ± 0.4	7.12 ± 0.5	9.98 ± 0.7	14.3 ± 1.1	21.14 ± 3.3
H-CBD-TER	21.67 ± 0.8	33.8 ± 4.4	59.65 ± 3.9	65.5 ± 7.1	79.7 ± 8.4	85.5 ± 5.5	49.55 ± 5.8	62.14 ± 7.5	73.4 ± 4.9	88.33 ± 8.1	91.2 ± 5.9	92.34 ± 5.5

**Table 4:** The antibacterial activity of tested hydrogels expressed as the diameter of the average inhibition zone (mm).

Test Microorganism	Zone of Inhibition [mm] (Mean ± SD)							
	H-CON		H-CBD		H-TER		H-CBD-TER	
	1.0%	5.0%	1.0%	5.0%	1.0%	5.0%	1.0%	5.0%
*Staphylococcus aureus*	nd	nd	8 ± 1	14 ± 1	10 ± 1	18 ± 2	12 ± 2	20 ± 2
*Staphylococcus epidermidis*	nd	nd	7 ± 1	10 ± 1	18 ± 3	24 ± 3	14 ± 1	22 ± 2
*Staphylococcus capitis*	nd	nd	11 ± 2	17 ± 2	10 ± 2	14 ± 2	14 ± 1	20 ± 1
*Micrococcus luteus*	nd	nd	8 ± 1	11 ± 1	10 ± 1	16 ± 3	15 ± 2	22 ± 2
*Propionibacterium acnes*	nd	nd	nd	6 ± 1	9 ± 1	13 ± 2	10 ± 1	15 ± 2
*Corynebacterium xerosis*	nd	nd	7 ± 1	11 ± 1	11 ± 1	16 ± 2	12 ± 1	17 ± 2
*Bacillus subtilis*	nd	nd	nd	nd	nd	nd	nd	nd
*Kocuria kristinae*	nd	nd	11 ± 1	16 ± 2	7 ± 1	9 ± 1	23 ± 2	34 ± 3
*Lactobacillus acidophilus*	nd	nd	nd	nd	nd	5 ± 1	nd	nd
*Candida albicans*	nd	nd	14 ± 1	22 ± 2	23 ± 12	33 ± 3	25 ± 3	36 ± 3
*Malassezia furfur*	nd	nd	nd	nd	12 ± 1	16 ± 1	11 ± 1	16 ± 1

nd—not detected.

## Data Availability

Data are contained within the article.

## Supplementary Materials

The following supporting information can be downloaded at: https://www.mdpi.com/article/10.3390/ijms25115934/s1.
