# Supplementary figures and images for: New Polymeric Hydrogels with Cannabidiol and α-Terpineol as Potential Materials for Skin Regeneration—Synthesis and Physicochemical and Biological Characterization
Source: Int J Mol Sci. 2024 May 29;25(11):5934. doi: 10.3390/ijms25115934 (PMC11173307; doi:10.3390/ijms25115934)

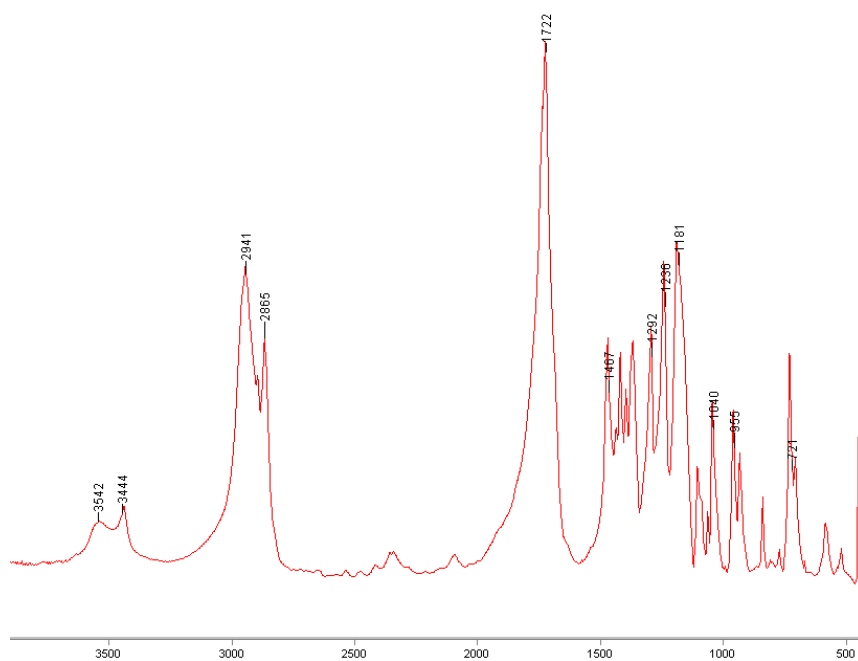

**Figure S3.** FT-IR spectrum of copolymers of CL and PEG (KBr).

Supplement: Supplementary file 1 [file ijms-25-05934-s001.zip › ijms-3024232-supplementary.pdf]
